# Parental Education, Own Education, and Cognitive Function in Middle-Aged and Older Adults

**DOI:** 10.1001/jamanetworkopen.2025.13036

**Published:** 2025-05-30

**Authors:** Shengyu Luo, Weiqing Chen, Wanting Hu, Harry H. X. Wang, Jinghua Li, Vivian Yawei Guo, David H. Rehkopf

**Affiliations:** 1 Department of Epidemiology, School of Public Health, Sun Yat-Sen University, Guangzhou, China; 2 School of Public Health, Sun Yat-Sen University, Guangzhou, China; 3 JC School of Public Health and Primary Care, Faculty of Medicine, The Chinese University of Hong Kong, Shatin, Hong Kong SAR, China; 4 Department of Biostatistics, School of Public Health, Sun Yat-Sen University, Guangzhou, Guangdong, China; 5 Department of Epidemiology and Population Health, Stanford School of Medicine, Stanford University, Stanford, California; 6 Division of Primary Care and Population Health, Department of Medicine, Stanford School of Medicine, Stanford University, Stanford, California; 7 Department of Pediatrics, Stanford School of Medicine, Stanford University, Stanford, California; 8 Department of Health Policy, Stanford School of Medicine, Stanford University, Stanford, California; 9 Department of Sociology, Stanford University, Stanford, California; 10 Center for Population Health Sciences, Stanford University, Stanford, California

## Abstract

**Importance:**

There is a lack of research examining the direct and independent associations of maternal and paternal education with later-life cognitive decline across diverse contexts.

**Objective:**

To evaluate the independent association of maternal and paternal education with cognitive decline over time among middle-aged and older adults in 4 countries, and to assess whether these associations are mediated by individuals’ own education.

**Design, Setting, and Participants:**

This multicohort study used data from 4 nationally representative cohorts, including the China Health and Retirement Longitudinal Study (CHARLS; 2011-2015), the Health and Retirement Study (HRS; 2010-2018) in the US, the English Longitudinal Study of Aging (ELSA; 2010-2018), and the Mexican Health and Aging Study (MHAS; 2012-2018). The study population consisted of participants aged 50 years or older with complete data on parental education, cognitive function, and covariates and without cognitive impairment at baseline. Statistical analysis was performed from November 1, 2023, to April 30, 2024.

**Exposures:**

Participants self-reported both parents’ education, which was further harmonized into 4 categories based on the 2011 International Standard Classification of Education (ie, less than primary, primary, lower secondary, and upper secondary or higher).

**Main Outcomes and Measures:**

The primary outcome was cognitive function, measured in 2 domains (ie, episodic memory and mental status). An overall cognitive state score was derived by combining scores from these 2 domains. All cognitive outcomes were standardized as *z* scores according to the baseline mean and SD within each cohort. Linear mixed-effects models were used to estimate the associations between parental education and cognitive decline over time. Generalized structural equation models were applied to examine the mediating role of participants’ own education in such associations.

**Results:**

This study included 7898 participants from China (mean [SD] age, 61.0 [7.4] years; 4141 men [52.4%]), 12 402 from the US (mean [SD] age, 64.8 [9.7] years; 5304 men [42.8%]), 6603 from England (mean [SD] age, 65.3 [8.2] years; 2915 men [44.1%]), and 9162 from Mexico (mean [SD] age, 63.4 [8.5] years; 3863 men [42.2%]). Compared with less than primary education, maternal education at the upper secondary level or higher was associated with slower rates of decline in cognitive state (β = 0.082 [95% CI, 0.035 to 0.129] SD per year in CHARLS, β = 0.025 [95% CI, 0.012 to 0.038] SD per year in HRS, and β = 0.040 [95% CI, 0.016 to 0.064] SD per year in ELSA). In MHAS, only primary maternal education was associated with slower decline in cognitive state (β = 0.010 [95% CI, 0.004 to 0.017] SD per year). Similarly, paternal education at the upper secondary level or higher, compared with less than primary, was associated with slower cognitive state decline in China (β = 0.042 [95% CI, 0.021 to 0.063] SD per year), the US (β = 0.027 [95% CI, 0.014 to 0.039] SD per year), and England (β = 0.044 [95% CI, 0.012 to 0.077] SD per year), whereas in Mexico, a significant association was observed for primary paternal education (β = 0.010 [95% CI, 0.004 to 0.017] SD per year). Participants’ own education significantly mediated the associations between parental education and annual decline in cognitive state in all cohorts, except for MHAS.

**Conclusions and Relevance:**

In this cohort study of middle-aged and older adults across 4 countries, both higher maternal and paternal education were generally associated with slower cognitive decline, and these associations were mediated by participants’ own education. These findings highlight the long-term relevance of parental education for offspring cognitive health across diverse cultural or socioeconomic contexts, and support the potential benefits of improving educational attainment to reduce intergenerational disparities in late-life cognitive health.

## Key Points

**Question:**

Are maternal and paternal education associated with cognitive declines in middle-aged and older adults, and do individuals’ own education mediate these associations?

**Findings:**

In this cohort study of 36 065 adults aged 50 years or older from China, the US, England, and Mexico, both higher maternal and paternal education were independently associated with slower cognitive decline, except in episodic memory domain in the US. These associations were mediated by individuals’ own education, except in Mexico.

**Meaning:**

These findings highlight the long-term relevance of parental education for offspring cognitive health and suggest that improving educational attainment may help reduce intergenerational disparities in late-life cognitive health.

## Teaser

This cohort study examines the association of maternal and paternal education with cognitive decline over time among middle-aged and older adults in 4 countries and assesses whether these associations are mediated by individuals’ own education.

## Introduction

Cognitive decline, often preceding dementia, presents an unprecedented global challenge as aging populations expand. It substantially impacts the quality of life and well-being of affected individuals and their families, drawing attention from researchers and policymakers worldwide.^1,2^ Considering the absence of effective treatment for late-stage dementia, identifying modifiable risk factors associated with cognitive decline before its progression to dementia is crucial.

Parental education is a key indicator of childhood resources and supports. Prior studies^3,4,5,6,7,8,9,10,11,12^ found that lower parental education is associated with poorer cognitive function and accelerated decline in middle-aged and older adults. However, limitations exist. First, parental education is often treated as a component of childhood socioeconomic status, making it challenging to identify its direct impacts.^3,4,7,8,9,10^ Second, the common practice of considering only the more educated parent may overlook the independent contributions of each parent, thus leading to an incomplete understanding of their separate associations with offspring’s cognitive function.^8,11,12^ Third, existing research has largely focused on single national contexts, limiting both the generalizability and the ability to compare findings across diverse cultural or socioeconomic settings. From the perspective of the International Classification of Functioning, Disability, and Health framework, inequalities in offspring cognitive health linked to parental education can be shaped by country-level and culture-level factors, such as differences in education systems and gender roles in parenting.^13^ When associations between parental education and offspring cognitive health remain consistent across heterogeneous contexts, it suggests that these associations are less likely to be driven by particular local confounding factors or any single country’s educational structures.^14^ Therefore, cross-national investigations are essential.

An individual’s own education also substantially contributes to cognitive health, with higher education linked to better cognitive performance and slower cognitive decline in later life.^15,16,17^ Moreover, research has consistently revealed a strong correlation between parents’ and their children’s educational levels, referred to as the intergenerational educational persistence.^18,19^ Therefore, an individual’s own education may mediate the associations between parental education and their cognitive function. Although some previous studies have supported this perspective,^5,6,8,20^ most were limited by cross-sectional design^6,8,20^ or small sample sizes.^5^ There is also a paucity of research separately examining how offspring’s education mediates the association of maternal vs paternal education with cognitive decline.

To address these gaps, we conducted a multicohort observational study with nationally representative data from China, the US, England, and Mexico. Our study aimed to (1) examine the longitudinal association of maternal and paternal education with cognitive decline in middle-aged and older adults across these 4 countries, and (2) explore whether participants’ own education mediated the association.

## Methods

### Study Design and Participants

This multicohort study used nationally representative data from 4 comparable cohorts, including the China Health and Retirement Longitudinal Study (CHARLS), the Health and Retirement Study (HRS), the English Longitudinal Study of Aging (ELSA), and the Mexican Health and Aging Study (MHAS). Detailed study designs and settings of these cohorts have been described previously.^21,22,23,24^ To ensure comparability, we included participants aged 50 years or older from waves 1 to 3 for CHARLS (2011-2015), waves 10 to 14 for HRS (2010-2018), waves 5 to 9 for ELSA (2010-2018), and waves 3 to 5 for MHAS (2012-2018).

The participant selection process for each cohort is outlined in eFigures 1 to 4 in Supplement 1. Participants with complete baseline data on parental education, cognitive function, and covariates were included. Consistent with previous studies,^25^ we excluded those with memory-related diseases (dementia and Parkinson disease) or a baseline *z* score of cognitive state less than 10th percentile to minimize recall bias. Participants with cognition data in at least 1 follow-up wave were included, whereas those lost to all follow-up waves or without cognition data in all waves were excluded.

Ethical approvals were obtained from the respective institutional review boards, with informed consent obtained from each participant. No further ethical approval was needed for using publicly available deidentified data. This study was reported in accordance with Strengthening the Reporting of Observational Studies in Epidemiology (STROBE) reporting guidelines.

### Measurement of Cognitive Function

Cognitive function was assessed at baseline and follow-up visits in 2 domains: episodic memory and mental status. Domain-specific scores were computed by summing the scores of the corresponding cognitive tests. An overall cognitive state score was then calculated by combining these 2 domain scores. Detailed descriptions of the cognitive assessment tasks for each cohort are elaborated in eMethods and eTable 1 in Supplement 1. To facilitate comparisons, standardized *z* scores were constructed by subtracting the baseline mean and dividing by the SD. All analyses used these standardized *z* scores as a proxy of cognitive function, with positive values indicating better performance than the mean population and negative values indicating poorer performance.

### Measurement of Parental and Own Education

Participants self-reported both parental and own educational levels. In CHARLS, HRS, and MHAS, parental education was recorded as the highest level of education attained, whereas in ELSA, it was based on the age at which education was completed. To ensure consistency across cohorts, parental education was harmonized into 4 categories: less than primary, primary, lower secondary, and upper secondary or higher, following the 2011 International Standard Classification of Education.^26^ Participants’ own education was classified into 5 categories: less than primary, primary, lower secondary, upper secondary, and postsecondary education. The detailed classification for each cohort was presented in eTables 2 and 3 in Supplement 1.

### Covariates

The covariates included self-reported baseline age, sex, and being part of a minoritized racial or ethnic group. Race and ethnicity were included as they were known to be associated with education attainment and cognitive function.^27,28^ Participants were classified as Han or minoritized ethnic group in CHARLS and as White or all other races in HRS and ELSA. MHAS did not collect racial or ethnic information; thus, this covariate was not adjusted for.

### Statistical Analysis

Data were analyzed from November 1, 2023 to April 30, 2024. All analyses were conducted separately for each cohort. Baseline characteristics for each cohort were reported as mean (SD) for continuous variables and frequency (percentage) for categorical variables.

The association between parental education and cognitive decline was examined using multivariable linear mixed-effects models with random intercepts and slopes to account for repeated cognitive measures and participant-specific cognitive trajectories. Models included parental education, follow-up time (year), a quadratic term for follow-up time, an interaction term (parental education by follow-up time), and covariates. When examining maternal education, paternal education was included as a covariate, and vice versa. The quadratic term was incorporated because it could improve model fit and capture potential nonlinear cognitive trajectories. Furthermore, mixed-effects Tobit models were specifically applied to the mental status domain in CHARLS and HRS to address ceiling effects, because more than 15% of observations reached the highest possible score.^29,30^ Results were presented as β coefficients with 95% CIs. To address the impact of missing data, we compared baseline characteristics between included and excluded participants within each cohort, and reanalyzed the associations using imputed datasets generated via multiple imputation by chained equations.

In mediation analyses, parental education was dichotomized as low (less than primary) and high (primary or higher) to facilitate model interpretation. Participants’ own education was similarly categorized as low (less than upper secondary) and high (upper secondary or higher). These classifications were based on the rationale that both parental education at or above the primary level and participants’ education at or above the upper secondary level were associated with slower decline rates in cognitive state across cohorts, although statistical significance was not always reached (eTable 4 in Supplement 1). The mediating role of participants’ own education on the rates of decline in cognitive state was evaluated by generalized structural equation models with individual-specific random intercepts and slopes.^31^ Bootstrapping with 2000 samples was performed to obtain bias-corrected 95% CI. All generalized structural equation models were adjusted for the same set of covariates as the linear mixed-effects models.

Statistical analyses were performed with Stata statistical software version 17.0 (StataCorp). A 2-tailed *P* < .05 indicated statistical significance.

## Results

This cohort study included 36 065 participants aged 50 years or older: 7898 participants from CHARLS (baseline mean [SD] age, 61.0 [7.4] years; 4141 men [52.4%] and 3757 women [47.6%]), 12 402 participants from HRS (mean [SD] age, 64.8 [9.7] years; 5304 men [42.8%] men and 7098 women [57.2%]), 6603 participants from ELSA (mean [SD] age, 65.3 [8.2] years; 2915 men [44.1%] men and 3688 women [55.9%]), and 9162 participants from MHAS (mean [SD] age, 63.4 [8.5] years; 3863 men [42.2%] and 5299 women [57.8%]) (Table 1). In CHARLS and MHAS, most participants reported that their mothers (CHARLS, 7204 participants [91.2%]; MHAS, 4489 participants [49.0%]) and fathers (CHARLS, 4693 participants [59.4%]; MHAS, 3926 participants [42.9%]) had less than primary education, whereas more than 70% of HRS and ELSA participants had parents with at least lower secondary education. Similarly, for participants’ own educational levels, a large proportion in HRS (6892 participants [55.8%]) and ELSA (3233 participants [49.1%]) attained postsecondary education, compared with much lower rates in CHARLS (158 participants [2.0%]) and MHAS (993 participants [10.9%]).

**Table 1.** Baseline Characteristics of Participants in the CHARLS, HRS, ELSA, and MHAS Cohorts

Characteristic	Participants, No. (%) (N = 36 065)			
	CHARLS (n = 7898)	HRS (n = 12 402)	ELSA (n = 6603)^a^	MHAS (n = 9162)^b^
Age, mean (SD), y	61.0 (7.4)	64.8 (9.7)	65.3 (8.2)	63.4 (8.5)
Sex				
Male	4141 (52.4)	5304 (42.8)	2915 (44.1)	3863 (42.2)
Female	3757 (47.6)	7098 (57.2)	3688 (55.9)	5299 (57.8)
Minoritized racial or ethnic group				
Yes	550 (7.0)	2743 (22.1)	155 (2.3)	NA
No	7348 (93.0)	9659 (77.9)	6448 (97.7)	NA
Own educational level				
Less than primary	1793 (22.7)	30 (0.2)	19 (0.3)	1240 (13.6)
Primary	3552 (45.0)	361 (2.9)	NA	4713 (51.6)
Lower secondary	1552 (19.7)	508 (4.1)	1732 (26.3)	1737 (19.0)
Upper secondary	840 (10.6)	4560 (36.9)	1605 (24.4)	452 (4.9)
Postsecondary	158 (2.0)	6892 (55.8)	3233 (49.1)	993 (10.9)
Maternal educational level				
Less than primary	7204 (91.2)	408 (3.3)	91 (1.4)	4489 (49.0)
Primary	592 (7.5)	1550 (12.5)	NA	4221 (46.1)
Lower secondary	52 (0.7)	2459 (19.8)	4703 (71.2)	452 (4.9)
Upper secondary or higher	50 (0.6)	7985 (64.4)	1809 (27.4)	NA
Paternal educational level				
Less than primary	4693 (59.4)	425 (3.4)	51 (0.8)	3926 (42.9)
Primary	2688 (34.0)	2110 (17.0)	NA	4556 (49.7)
Lower secondary	275 (3.5)	2800 (22.6)	4813 (72.9)	680 (7.4)
Upper secondary or higher	242 (3.1)	7067 (57.0)	1739 (26.3)	NA

Abbreviations: CHARLS, the China Health and Retirement Longitudinal Study; ELSA, the English Longitudinal Study of Aging; HRS, the Health and Retirement Study; MHAS, the Mexican Health and Aging Study; NA, not applicable.

^a^ Available options of parental and participants’ own education in ELSA did not allow for a distinction between primary and lower secondary education.

^b^ Available options of parental education in MHAS did not allow for a distinction between lower secondary and upper secondary education. No racial or ethnic information was available in MHAS.

As shown in Table 2 and Table 3, both higher maternal and paternal education were generally associated with better baseline cognitive state and slower decline rates across cohorts. Specifically, compared with participants whose mothers had less than primary education, those whose mothers attained upper secondary education or higher exhibited significantly slower decline in cognitive state (β = 0.082 [95% CI, 0.035 to 0.129] SD per year for CHARLS; β = 0.025 [95% CI, 0.012 to 0.038] SD per year for HRS; β = 0.040 [95% CI, 0.016 to 0.064] SD per year for ELSA) (Table 2). For MHAS, only primary maternal education was significantly associated with slower decline in cognitive state (β = 0.010 [95% CI, 0.004 to 0.017] SD per year). Similarly, compared with participants of fathers with less than primary education, having a father with upper secondary education or higher was associated with slower decline in cognitive state (β = 0.042 [95% CI, 0.021 to 0.063] SD per year for CHARLS; β = 0.027 [95% CI, 0.014 to 0.039] SD per year for HRS, β = 0.044 [95% CI, 0.012 to 0.077] SD per year for ELSA) (Table 3). For MHAS, only primary paternal education was significantly associated with slower cognitive state decline over time (β = 0.010 [95% CI, 0.004 to 0.017] SD per year). Consistent patterns for both maternal and paternal education were observed in the episodic memory and mental status domains across cohorts, except for episodic memory in HRS (eTables 5 and 6 in Supplement 1).

**Table 2.** Association Between Maternal Education and Cognitive State

Variable	β coefficient (95% CI)^a^			
	CHARLS	HRS	ELSA	MHAS
Maternal educational level, SD				
Less than primary	0 [Reference]	0 [Reference]	0 [Reference]	0 [Reference]
Primary	0.141 (0.077 to 0.206)	0.060 (−0.033 to 0.154)	NA	0.221 (0.184 to 0.258)
Lower secondary	0.193 (−0.004 to 0.391)	0.158 (0.061 to 0.254)	0.038 (−0.131 to 0.206)	0.491 (0.403 to 0.580)
Upper secondary or higher	0.272 (0.070 to 0.473)	0.231 (0.136 to 0.326)	0.158 (−0.014 to 0.330)	NA
Maternal educational level × time, SD/y				
Less than primary	0 [Reference]	0 [Reference]	0 [Reference]	0 [Reference]
Primary	0.033 (0.017 to 0.049)	0.012 (−0.002 to 0.026)	NA	0.010 (0.004 to 0.017)
Lower secondary	0.076 (0.031 to 0.121)	0.019 (0.005 to 0.032)	0.026 (0.002 to 0.049)	−0.001 (−0.017 to 0.015)
Upper secondary or higher	0.082 (0.035 to 0.129)	0.025 (0.012 to 0.038)	0.040 (0.016 to 0.064)	NA

Abbreviations: CHARLS, the China Health and Retirement Longitudinal Study; ELSA, the English Longitudinal Study of Aging; HRS, the Health and Retirement Study; MHAS, the Mexican Health and Aging Study; NA, not applicable.

^a^ Models were adjusted for participants’ baseline age, sex, minoritized racial or ethnic group, and paternal educational level. A total of 7898 participants with complete data on covariates were included in CHARLS, 12402 in HRS, 6603 in ELSA, and 9162 in MHAS.

**Table 3.** Association Between Paternal Education and Cognitive State

Variable	β coefficient (95% CI)^a^			
	CHARLS	HRS	ELSA	MHAS
Paternal educational level, SD				
Less than primary	0 [Reference]	0 [Reference]	0 [Reference]	0 [Reference]
Primary	0.205 (0.169 to 0.240)	0.177 (0.087 to 0.268)	NA	0.181 (0.144 to 0.218)
Lower secondary	0.183 (0.090 to 0.276)	0.198 (0.104 to 0.292)	0.155 (−0.055 to 0.366)	0.499 (0.424 to 0.575)
Upper secondary or higher	0.288 (0.190 to 0.387)	0.320 (0.227 to 0.413)	0.260 (0.047 to 0.473)	NA
Paternal educational level × time, SD/y				
Less than primary	0 [Reference]	0 [Reference]	0 [Reference]	0 [Reference]
Primary	0.019 (0.009 to 0.029)	0.014 (0.001 to 0.027)	NA	0.010 (0.004 to 0.017)
Lower secondary	0.015 (−0.008 to 0.038)	0.028 (0.015 to 0.041)	0.033 (0.001 to 0.066)	−0.003 (−0.017 to 0.010)
Upper secondary or higher	0.042 (0.021 to 0.063)	0.027 (0.014 to 0.039)	0.044 (0.012 to 0.077)	NA

Abbreviations: CHARLS, the China Health and Retirement Longitudinal Study; ELSA, the English Longitudinal Study of Aging; HRS, the Health and Retirement Study; MHAS, the Mexican Health and Aging Study; NA, not applicable.

^a^ Models were adjusted for participants’ baseline age, sex, minoritized racial or ethnic groups, and maternal educational level. A total of 7898 participants with complete data on covariates were included in CHARLS, 12402 in HRS, 6603 in ELSA, and 9162 in MHAS.

Compared with those excluded because of missing data or loss to follow-up, included participants were generally younger, less likely to be from minoritized racial and ethnic groups, and more likely to have highly educated parents and higher own educational level, with some exceptions in CHARLS (eTables 7 to 10 in Supplement 1). Results from imputed datasets were consistent with main analyses, showing that higher parental education was associated with slower cognitive decline across domains and cohorts (eTables 11 and 12 in Supplement 1).

As shown in Table 4, the association between maternal education and changes in cognitive state was significantly mediated by participants’ own education in all cohorts, except for MHAS. Specifically, the β coefficients of indirect effects through participants’ own education were 0.025 (95% CI, 0.018 to 0.031) in CHARLS, 0.047 (95% CI, 0.032 to 0.060) in HRS, 0.016 (95% CI, 0.011 to 0.022) in ELSA, and 0.010 (95% CI, −0.004 to 0.025) in MHAS. Similarly, participants’ own education also significantly mediated the association between paternal education and changes in cognitive state in all cohorts, except for MHAS. The β coefficients of indirect effects through participants’ own education were 0.020 (95% CI, 0.012 to 0.027) in CHARLS, 0.063 (95% CI, 0.047 to 0.087) in HRS, 0.022 (95% CI, 0.015 to 0.031) in ELSA, and 0.009 (95% CI, −0.0004 to 0.014) in MHAS.

**Table 4.** Mediating Role of Participants’ Own Education in the Association Between Parental Education and Rates of Decline in Cognitive State Over Time

Variable	Path coefficients, β (95% CI), SD/y^a^		
	Direct effect	Indirect effect through participants’ own education	Total effect
Maternal education^b^			
CHARLS	0.034 (0.014 to 0.045)	0.025 (0.018 to 0.031)	0.060 (0.029 to 0.072)
HRS	0.008 (0.001 to 0.022)	0.047 (0.032 to 0.060)	0.055 (0.046 to 0.072)
ELSA	0.027 (0.015 to 0.038)	0.016 (0.011 to 0.022)	0.043 (0.028 to 0.056)
MHAS	0.007 (0.001 to 0.014)	0.010 (−0.004 to 0.025)	0.018 (0.004 to 0.032)
Paternal education^c^			
CHARLS	0.018 (0.009 to 0.023)	0.020 (0.012 to 0.027)	0.038 (0.025 to 0.049)
HRS	0.010 (0.003 to 0.013)	0.063 (0.047 to 0.087)	0.073 (0.060 to 0.090)
ELSA	0.032 (0.014 to 0.059)	0.022 (0.015 to 0.031)	0.055 (0.038 to 0.080)
MHAS	0.007 (0.0001 to 0.011)	0.009 (−0.0004 to 0.014)	0.016 (0.008 to 0.023)

Abbreviations: CHARLS, the China Health and Retirement Longitudinal Study; ELSA, the English Longitudinal Study of Aging; HRS, the Health and Retirement Study; MHAS, the Mexican Health and Aging Study.

^a^ A total of 7895 participants with complete data on covariates were included in CHARLS, 12351 in HRS, 6589 in ELSA, and 9134 in MHAS.

^b^ Models were adjusted for participants’ baseline age, sex, minoritized racial or ethnic groups, and paternal educational level. Low maternal education (less than primary) was the reference group.

^c^ Models were adjusted for participants’ baseline age, sex, minoritized racial or ethnic groups, and maternal educational level. Low paternal education (less than primary) was the reference group.

## Discussion

In this multicohort observational study, we identified that, after mutually adjusting for each other, both higher maternal and paternal educational levels were associated with slower cognitive decline among middle-aged and older adults across countries with diverse cultural and socioeconomic contexts, except for the episodic memory domain in HRS. Furthermore, participants’ own education significantly mediated the associations between parental education and declines in cognitive state, except in MHAS.

The exact mechanisms linking parental education to later-life cognitive health remain incompletely understood. The selective-elimination hypothesis suggests that childhood environmental inputs influence neural structure and functionality by selectively pruning underused synaptic connections.^32^ Parents with low education may be less able to provide cognitively stimulating environments,^33,34^ and thus synaptic connections that are rarely activated might be pruned away over time.^35,36,37^ This process could result in fewer synaptic connections and less-efficient neural networks. Over time, such deficits may contribute to poorer cognitive function and increased susceptibility to cognitive decline in later life.^35^ Furthermore, the prenatal period and early childhood are critical for brain development.^38^ Children of less-educated parents are more likely to face adverse conditions, such as malnutrition^39,40^ and increased exposure to infections and diseases.^38^ These early-life disadvantages could disrupt normal brain growth and development, potentially leading to long-term impairments in cognitive function.^38,41^

We identified that both higher maternal and paternal education were associated with slower cognitive decline in middle-aged and older adults after mutual adjustment, underscoring the crucial role both parents play in shaping offspring cognitive function. Mothers, who are often the primary caregivers, typically engage more directly in their children’s daily lives.^42^ A highly educated mother can offer high-quality interactions, provide nutritious diets, and expose the child to a variety of cognitive stimuli.^43,44^ These factors contribute to enhanced neural development in early stages, which is beneficial for cognitive health in later life.^44^ On the other hand, fathers typically are more involved in external work, and their educational level often reflects the resources and supports available to the child and the family.^42^ Children of less-educated fathers may face more challenging childhood circumstances, such as poor living conditions and nutritional deficiencies, which can negatively affect children’s brain development and lead to poor cognitive function in later life.^25^ Notably, we observed cross-national differences in the relative effect sizes of maternal and paternal education on cognitive decline. In CHARLS (China), maternal education showed a relatively larger effect size than paternal education, whereas in HRS (the US), ELSA (England), and MHAS (Mexico), the magnitudes were more comparable between parents. This pattern may reflect variations in caregiving roles across cultural contexts. In traditional Chinese households, particularly among older generations, mothers often play a more prominent role in raising children, while fathers usually adopt a more distant and overarching role in child-rearing.^45^ These culturally rooted caregiving dynamics may help explain the relatively stronger role of maternal education observed in the Chinese cohort.

This study further extends existing research by revealing the potential mediating role of an individual’s own education in the association between parental education and cognitive decline. This observation aligns with previous cross-sectional studies.^5,6,8,20^ Parents with higher education typically provide more resources and supports for their children, thereby leading to better developmental outcomes, including higher educational achievement.^20,40^ In turn, higher education exposes individuals to diverse cognitive stimuli, promoting the development of neural networks that contribute to cognitive reserve. This reserve plays a crucial role in counteracting neuropathological changes and cognitive decline.^17^ Moreover, education equips individuals with essential health literacy and problem-solving skills that are beneficial for cognitive maintenance.^46^ Higher education also increases employment opportunities, income, and job stability, which provide additional material and psychosocial resources that support cognitive health.^47^ Therefore, the mediating role of participants’ own education is plausible.

Overall, although our findings are observational in nature, they have important implications. First, by examining maternal and paternal education directly while mutually adjusting for each other, we identified significant associations between both parents’ education and offspring cognitive function. These findings underscore the need to consider the independent contributions of both maternal and paternal education in cognitive aging research. They also indicate that improving parental educational attainment may bring intergenerational benefits by enhancing not only life opportunities but also the cognitive health for the next generation. Second, by identifying the mediating role of participants’ own education, our study implies that promoting educational opportunities for children of less-educated parents may help reduce the intergenerational impacts of low parental education and mitigate its long-term consequences for cognitive health. Third, by analyzing data from 4 countries with diverse educational and sociocultural contexts, we demonstrated that the observed associations were generally consistent across settings, rather than confined to a single population. This cross-national perspective enhanced the robustness and generalizability of our findings and supported the broader relevance of parental education as an important determinant of cognitive health in later life.

### Strengths and Limitations

The strengths of our study included the use of nationally representative, cross-cultural data from 4 countries, which enhanced the robustness and generalizability of the findings across diverse contexts. The longitudinal design allowed us to examine the association of parental education with cognitive decline over time. Furthermore, mutual adjustment for maternal and paternal education in the model reduced potential bias from assortative mating.^48,49^ Finally, we identified the mediating role of participants’ own education, providing insight into pathways linking parental education to later-life cognitive decline.

Nevertheless, several limitations deserve further discussion. First, as this study is observational, causal inferences cannot be established. However, the consistent results across 4 diverse cohorts support the validity of our findings and highlight the need for further research to confirm the conclusions. Second, a substantial proportion of participants was excluded due to missing data or loss to follow-up, potentially limiting the generalizability of our findings. Additionally, although multiple imputation analysis produced results consistent with the main analyses, significant differences between excluded and analytic samples in certain characteristics raised concerns about internal validity. These issues should be carefully considered when interpreting the results. Third, reliance on self-reported parental and own education may introduce recall bias. However, excluding participants with memory-related diseases or extremely low baseline cognitive scores may have mitigated this concern. Fourth, due to data limitations, we were unable to account for potential confounders that occurred prior to parental education and cognitive function, such as genetic predisposition.^50,51^ As a result, residual confounding cannot be ruled out.

## Conclusions

In this multicohort study of middle-aged and older adults across 4 countries, both higher maternal and paternal education were generally associated with slower cognitive decline over time, and these associations were mediated by participants’ own education. These findings suggested that both parents’ education had long-term implication for offspring cognitive health and highlighted the potential of improving educational attainment across diverse contexts to reduce intergenerational disparities in later-life cognitive health.

## Notes

Supplement 1.
eMethods.
**eFigure 1.** Flowchart of participant selection in CHARLS **eFigure 2.** Flowchart of participant selection in HRS **eFigure 3.** Flowchart of participant selection in ELSA **eFigure 4.** Flowchart of participant selection in MHAS **eTable 1.** Measurement of cognitive function in CHARLS, HRS, ELSA, and MHAS **eTable 2.** Harmonized parental educational categories in each study according to the 2011 International Standard Classification of Education **eTable 3.** Harmonized participants’ own educational categories in each study according to the 2011 International Standard Classification of Education **eTable 4.** The association of participants’ own education with cognitive state **eTable 5.** The association of maternal education with episodic memory and mental status **eTable 6.** The association of paternal education with episodic memory and mental status **eTable 7.** Comparison of baseline characteristics between the analytic and excluded participants in CHARLS **eTable 8.** Comparison of baseline characteristics between the analytic and excluded participants in HRS **eTable 9.** Comparison of baseline characteristics between the analytic and excluded participants in ELSA **eTable 10.** Comparison of baseline characteristics between the analytic and excluded participants in MHAS **eTable 11.** The association between maternal education and cognitive function with imputed dataset **eTable 12.** The association between paternal education and cognitive function with imputed dataset
eReferences

Supplement 2.
Data Sharing Statement
