# Supplementary material for: Parental Education, Own Education, and Cognitive Function in Middle-Aged and Older Adults
Source: JAMA Netw Open. 2025 May 30;8(5):e2513036. doi: 10.1001/jamanetworkopen.2025.13036 (PMC12125642; doi:10.1001/jamanetworkopen.2025.13036)
Supplement: Supplement 1. — eMethods. eFigure 1. Flowchart of participant selection in CHARLS eFigure 2. Flowchart of participant selection in HRS eFigure 3. Flowchart of participant selection in ELSA eFigure 4. Flowchart of participant selection in MHAS eTable 1. Measurement of cognitive function in CHARLS, HRS, ELSA, and MHAS eTable 2. Harmonized parental educational categories in each study according to the 2011 International Standard Classification of Education eTable 3. Harmonized participants’ own educational categories in each study according to the 2011 International Standard Classification of Education eTable 4. The association of participants’ own education with cognitive state eTable 5. The association of maternal education with episodic memory and mental status eTable 6. The association of paternal education with episodic memory and mental status eTable 7. Comparison of baseline characteristics between the analytic and excluded participants in CHARLS eTable 8. Comparison of baseline characteristics between the analytic and excluded participants in HRS eTable 9. Comparison of baseline characteristics between the analytic and excluded participants in ELSA eTable 10. Comparison of baseline characteristics between the analytic and excluded participants in MHAS eTable 11. The association between maternal education and cognitive function with imputed dataset eTable 12. The association between paternal education and cognitive function with imputed dataset eReferences [file jamanetwopen-e2513036-s001.pdf]

Supplemental Online Content

Luo S, Chen W, Hu W, et al. Parental education, own education, and cognitive function in middle-aged and older adults. *JAMA Netw Open*. 2025;8(5):e2513036. doi:10.1001/jamanetworkopen.2025.13036

eMethods

eFigure 1. Flowchart of participant selection in CHARLS

eFigure 2. Flowchart of participant selection in HRS

eFigure 3. Flowchart of participant selection in ELSA

eFigure 4. Flowchart of participant selection in MHAS

eTable 1. Measurement of cognitive function in CHARLS, HRS, ELSA, and MHAS

eTable 2. Harmonized parental educational categories in each study according to the 2011 International Standard Classification of Education

eTable 3. Harmonized participants' own educational categories in each study according to the 2011 International Standard Classification of Education

eTable 4. The association of participants' own education with cognitive state

eTable 5. The association of maternal education with episodic memory and mental status

eTable 6. The association of paternal education with episodic memory and mental status

eTable 7. Comparison of baseline characteristics between the analytic and excluded participants in CHARLS

eTable 8. Comparison of baseline characteristics between the analytic and excluded participants in HRS

eTable 9. Comparison of baseline characteristics between the analytic and

© 2025 Luo S et al. *JAMA Network Open*.

excluded participants in ELSA

eTable 10. Comparison of baseline characteristics between the analytic and excluded participants in MHAS

eTable 11. The association between maternal education and cognitive function with imputed dataset

eTable 12. The association between paternal education and cognitive function with imputed dataset

eReferences

This supplemental material has been provided by the authors to give readers additional information about their work.

© 2025 Luo S et al. *JAMA Network Open*.

eMethods

Assessment of cognitive function

Cognitive function was assessed at baseline and follow-up across two domains: episodic memory and mental status. Detailed descriptions of the assessment tasks are provided in eTable 1. Episodic memory was evaluated using immediate and delayed word recall tasks, with 10 words administered in CHARLS, HRS, and ELSA, and 8 words in MHAS. The episodic memory score was calculated as the average number of correctly recalled words from both tasks. The score ranged from 0 to 10 in CHARLS, HRS, and ELSA, and 0 to 8 in MHAS. The tasks included in mental status domain varied by cohort. In CHARLS, it encompassed tasks of orientation (score range: 0-5), serial 7s subtraction (score range: 0-5), and visuospatial ability (score range: 0-1). In HRS, it included tasks of orientation (score range: 0-4), serial 7s subtraction (score range: 0-5), and backward counting (score range: 0-2). In ELSA, it was assessed using tasks of orientation (score range: 0-4) and animal naming (score range: 0-100), while MHAS included orientation (score range: 0-3), animal naming (score range: 0-60), and visuospatial learning and recall (score range: 0-6) tasks. An overall cognitive state score was derived by summing scores from both domains. The total score for cognitive state ranged from 0 to 21 in CHARLS and HRS, from 0 to 114 in ELSA, and from 0 to 77 in MHAS. The reliability and validity of these cognitive tasks have been well-established in existing literature.[1-5] To facilitate cross-cohort comparisons, standardized z scores were computed for each domain and the overall cognitive state by subtracting the baseline mean and dividing by the standard deviation. These continuous z scores were used throughout the study as a proxy measure of cognitive function, with higher z scores indicating better cognitive performance.

© 2025 Luo S et al. *JAMA Network Open*.

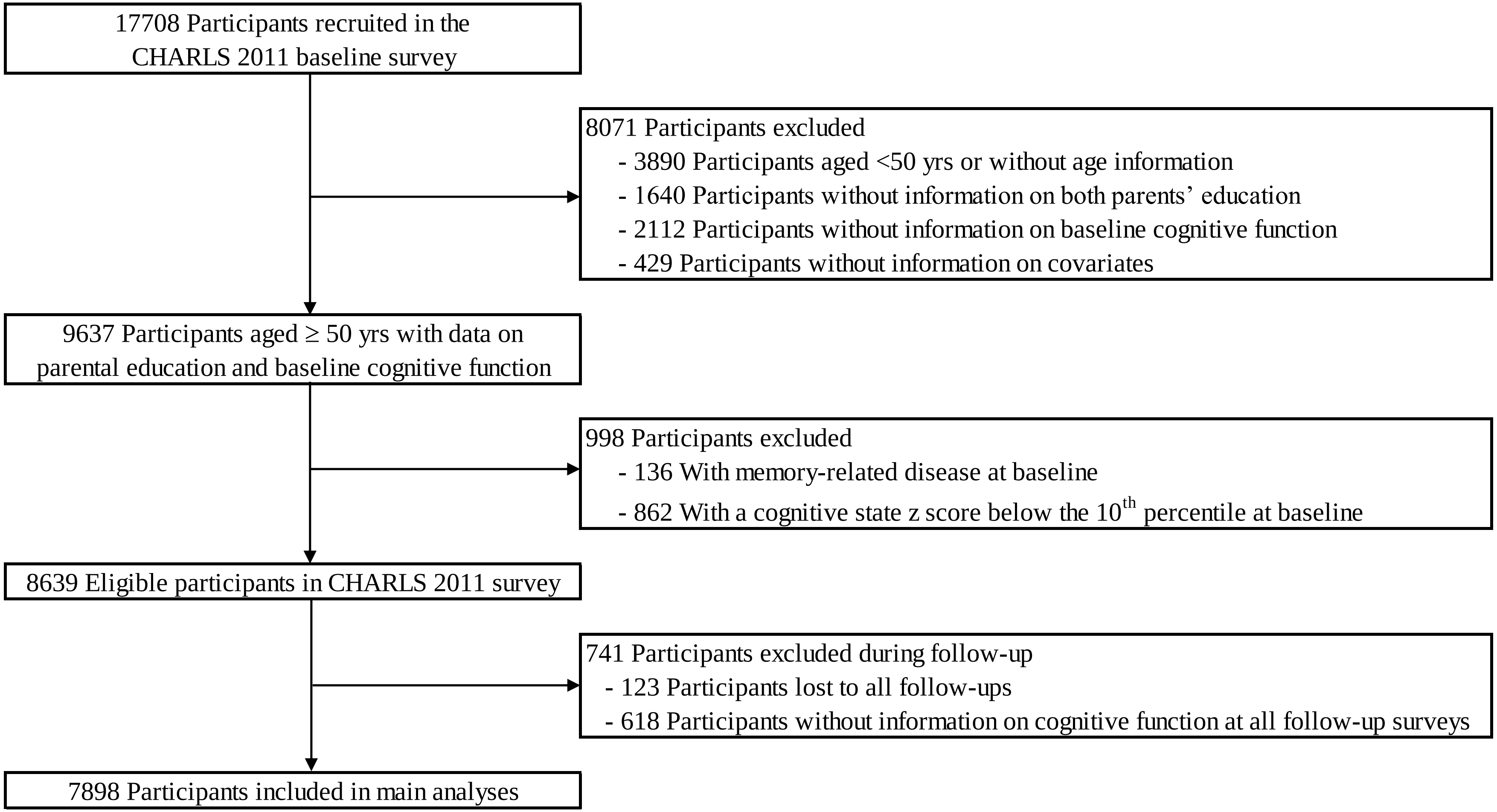

eFigure 1 Flowchart of participant selection in CHARLS

Abbreviation: CHARLS, the China Health and Retirement Longitudinal Study

© 2025 Luo S et al. *JAMA Network Open*.

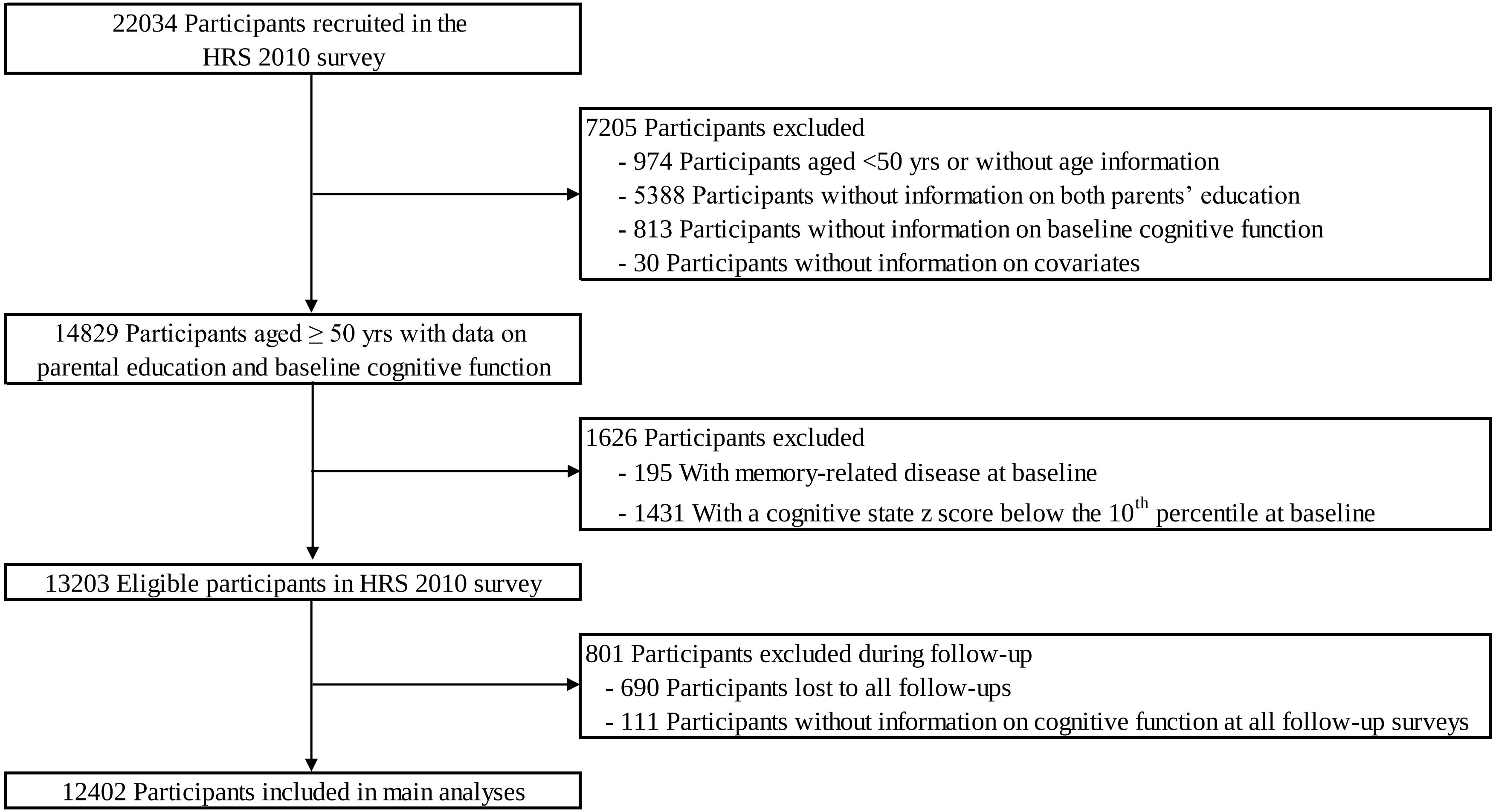

eFigure 2 Flowchart of participant selection in HRS

Abbreviation: HRS, the Health and Retirement Study

© 2025 Luo S et al. *JAMA Network Open*.

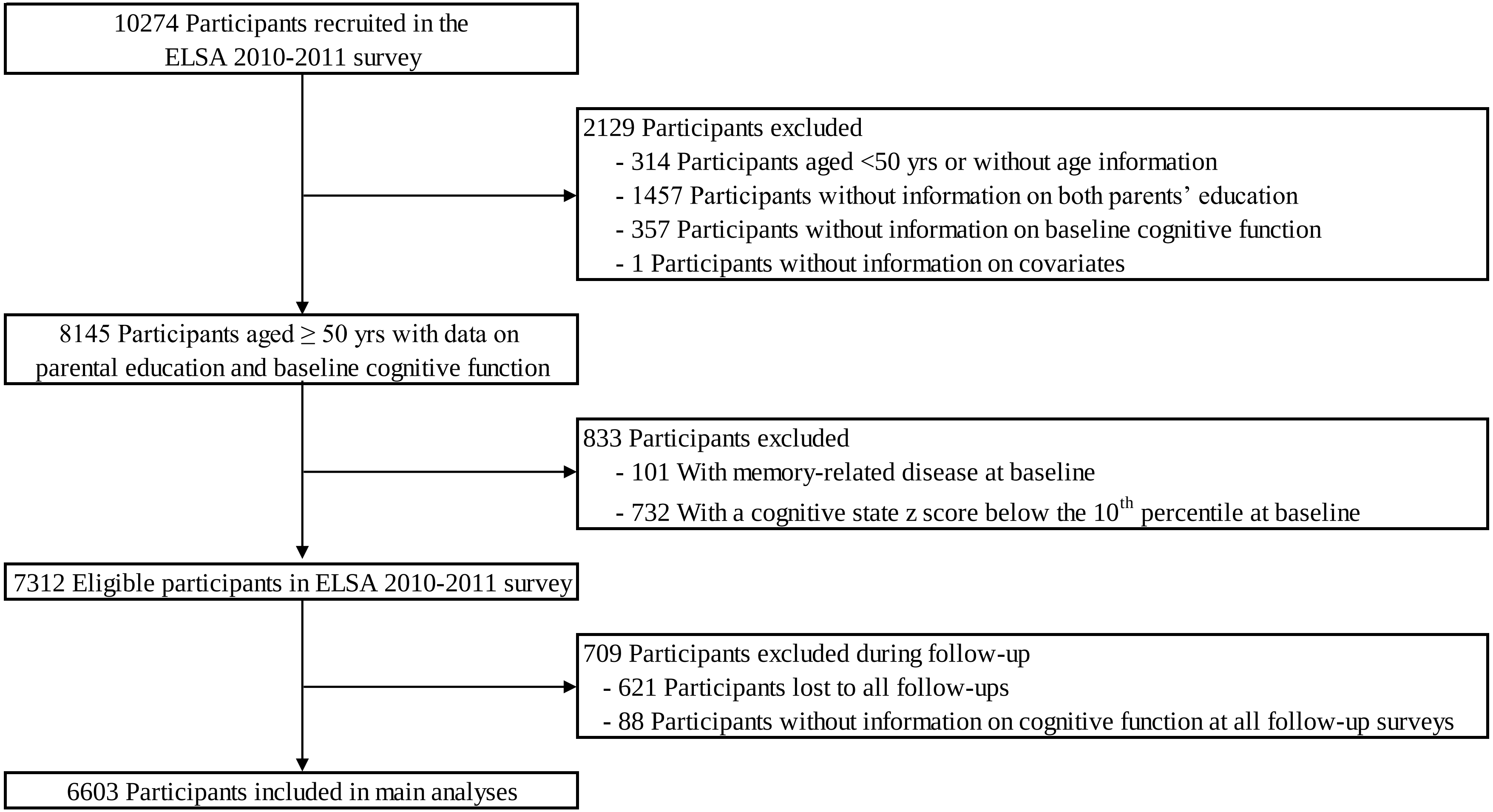

eFigure 3 Flowchart of participant selection in ELSA

Abbreviation: ELSA, the English Longitudinal Study of Ageing

© 2025 Luo S et al. *JAMA Network Open*.

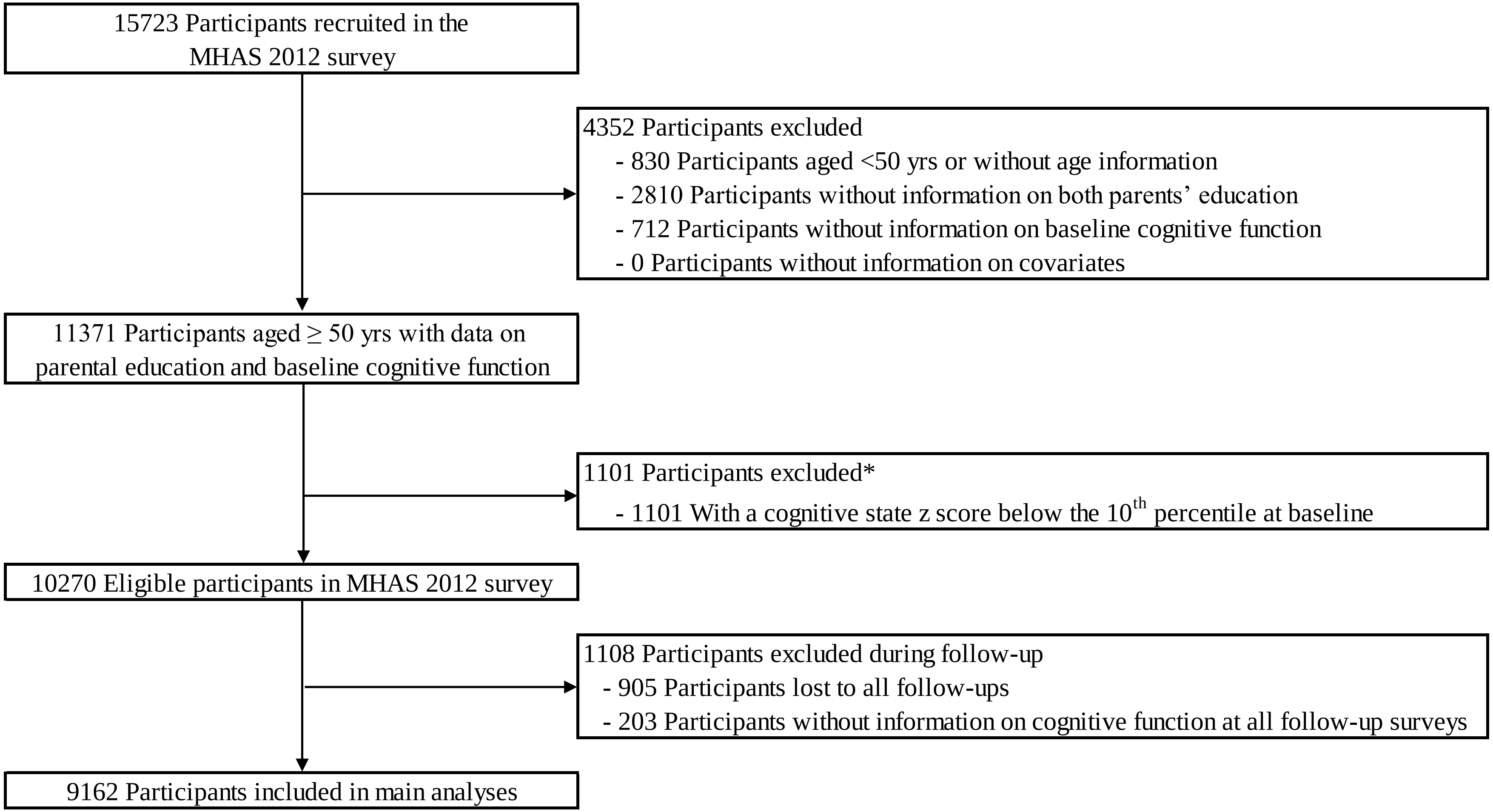

eFigure 4 Flowchart of participant selection in MHAS

*MHAS did not have information on memory-related diseases. Abbreviation: MHAS, the Mexican Health and Aging Study.

© 2025 Luo S et al. *JAMA Network Open*.

eTable 1 Measurement of cognitive function in CHARLS, HRS, ELSA, and MHAS

	Measurement			
	CHARLS	HRS	ELSA	MHAS
**Episodic memory**	**Immediate and delayed word recall (scores: 0-10 for CHARLS, HRS, and ELSA, and 0-8 for MHAS):** The participants were read a set of 10 words (in CHARLS, HRS, and ELSA) or 8 words (in MHAS) and were asked to recall the words as many as they can, in any order (Immediate recall). After conducting some other tests, the participants were again asked to recall the words they remembered (Delayed recall). Scores were generated by averaging the correct words in immediate and delayed recall.			
**Mental status**	**Orientation (scores: 0-5)**: The participants were asked to tell today's date (year, month, day, and day of the week) and the current season (spring, summer, fall, or winter).<br><br>**Serial 7s subtraction test (scores: 0-5)**: The participants were asked to calculate (1) What does 100 minus 7 equal? (2) And 7 from that? (3) And 7 from that? (4) And 7 from that? (5) And 7 from that?	**Orientation (scores: 0-4)**: The participants were asked to tell today's date (year, month, day, and day of the week).<br><br>**Serial 7s subtraction test (scores: 0-5)**: The participants were asked to calculate (1) What does 100 minus 7 equal? (2) And 7 from that? (3) And 7 from that? (4) And 7 from that? (5) And 7 from that?	**Orientation (scores: 0-4)**: The participants were asked to tell today's date (year, month, day, and day of the week).<br><br>**Animal naming (scores: 0-100)**: The participants were asked to name as many different animals as they can think of in one minute. Scores were summed as the number of different animals the participants told.	**Orientation (scores: 0-3)**: The participants were asked to tell today's date (year, month, and day).<br><br>**Animal naming (scores: 0-60)**: The participants were asked to name as many different animals as they can think of in one minute. Scores were summed as the number of different animals the participants told.<br><br>**Visuospatial learning and recall (scores: 0-6)**: The participants were requested to see and draw the following picture with two triangles and one square in 90 seconds (learning). After conducting some other

© 2025 Luo S et al. *JAMA Network Open*.

	Measurement			
	CHARLS	HRS	ELSA	MHAS
	**Visuospatial ability (scores: 0-1)**:<br>The participants were asked to see and draw a picture of two pentagons overlapped on the paper.	**Backward counting (scores: 0-2)**:<br>The participants were asked to count backwards for 10 continuous numbers as quickly as possible starting with the number 20. Two points were given for success in the first try, one point for success in the second try, and 0 for unsuccessful counting in both tries.		tests, the participants were again asked to draw the picture as they remembered (recall). Participants were given 6 points in each try if they drew the presence and the position of triangles and square correctly. Average scores for learning and recall tests were generated as final scores.<br>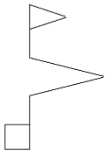

Abbreviations: CHARLS, the China Health and Retirement Longitudinal Study; HRS, the Health and Retirement Study; ELSA, the English Longitudinal Study of Ageing; MHAS, the Mexican Health and Aging Study

© 2025 Luo S et al. *JAMA Network Open*.

eTable 2 Harmonized parental educational categories in each study according to the 2011 International Standard Classification of Education

Variables	ISCED attainment levels	Measurement			
		CHARLS	HRS	ELSA[a]	MHAS[b]
Parental education	Less than primary	No formal education (illiterate)	No formal education	Never went to school	None
	Primary	Did not finish primary school;<br>Sishu/home school;<br>Elementary school	1-6 Grades	-	Some elementary;<br>Completed elementary
	Lower secondary	Middle school	7-9 Grades	Age finished continuous full-time education at 14 or under	Beyond elementary
	Upper secondary or higher	High school;<br>Vocational school;<br>Two-/Three-Year College/Associate degree;<br>Four-Year College/Bachelor's degree;<br>Master's degree;<br>Doctoral degree/Ph.D	10-12 High school;<br>13-15 Some college;<br>16 College grad;<br>17 Post college (17+ years)	Age finished continuous full-time education at 15 or over	-

[a] Available options of parental education in ELSA did not allow for a distinction between primary and lower secondary education.

[b] Available options of parental education in MHAS did not allow for a distinction between lower secondary and upper secondary education.

Abbreviations: ISCED, International Standard Classification of Education; CHARLS, the China Health and Retirement Longitudinal Study; HRS, the Health and Retirement Study; ELSA, the English Longitudinal Study of Ageing; MHAS, the Mexican Health and Aging Study

© 2025 Luo S et al. *JAMA Network Open*.

eTable 3 Harmonized participants' own educational categories in each study according to the 2011 International Standard Classification of Education

Variables	ISCED attainment levels	Measurement			
		CHARLS	HRS	ELSA[a]	MHAS
Participants' own education	Less than primary	No formal education (illiterate)	No formal education	Never went to school	0 year of schooling
	Primary	Did not finish primary school;<br>Sishu/home school;<br>Elementary school	1-6 Grades	-	1-6 years of schooling
	Lower secondary	Middle school	7-9 Grades	No qualification;<br>Foreign/undetermined qualification & Age finished continuous full-time education at 14 or under	7-9 years of schooling
	Upper secondary	High school;<br>Vocational school	10-12 High school	NVQ1/CSE other grade equivalent;<br>NVQ2/GCE O level equivalent;<br>Foreign/undetermined qualification & Age finished continuous full-time education at 15, 16, 17, or 18	10-12 years of schooling
	Postsecondary	Two-/Three-Year College/Associate degree;<br>Four-Year College/Bachelor's degree;<br>Master's degree;<br>Doctoral degree/Ph.D	13-15 Some college;<br>16 College grad;<br>17 Post college (17+ years)	NVQ3/GCE A level equivalent;<br>Higher education below degree;<br>NVQ4/NVQ5/degree or equivalent;<br>Foreign/undetermined qualification & Age finished continuous full-time education at 19 or over	More than 12 years of schooling

[a] Available options of participants' own education in ELSA did not allow for a distinction between primary and lower secondary education.

Abbreviations: ISCED, International Standard Classification of Education; CHARLS, the China Health and Retirement Longitudinal Study; HRS, the Health and Retirement Study; ELSA, the English Longitudinal Study of Ageing; MHAS, the Mexican Health and Aging Study; NVQ, National Vocational Qualifications; CSE, Certificate of Secondary Education; GCE, General Certificate of Education

© 2025 Luo S et al. *JAMA Network Open*.

eTable 4 The association of participants' own education with cognitive state

	β Coefficients (95% CI)[a]			
	**CHARLS**	**HRS**	**ELSA**	**MHAS**
**Participants' own education, SD**				
< primary	0 [Reference]	0 [Reference]	0 [Reference]	0 [Reference]
primary	0.596 (0.558, 0.635)	-0.002 (-0.265, 0.261)	-	0.313 (0.268, 0.358)
lower secondary	0.932 (0.885, 0.979)	-0.012 (-0.275, 0.252)	-0.243 (-0.570, 0.083)	0.725 (0.670, 0.780)
upper secondary	1.115 (1.059, 1.172)	0.249 (-0.009, 0.508)	-0.080 (-0.406, 0.247)	0.827 (0.748, 0.905)
postsecondary	1.304 (1.195, 1.412)	0.516 (0.258, 0.775)	0.182 (-0.143, 0.508)	1.033 (0.968, 1.098)
**Participants' own education × time, SD/year**				
< primary	0 [Reference]	0 [Reference]	0 [Reference]	0 [Reference]
primary	0.054 (0.043, 0.066)	0.016 (-0.033, 0.065)	-	0.032 (0.022, 0.041)
lower secondary	0.067 (0.053, 0.080)	0.021 (-0.028, 0.069)	-0.009 (-0.065, 0.047)	0.032 (0.021, 0.043)
upper secondary	0.078 (0.061, 0.095)	0.042 (-0.005, 0.089)	0.018 (-0.038, 0.074)	0.035 (0.018, 0.051)
postsecondary	0.084 (0.050, 0.118)	0.054 (0.007, 0.101)	0.017 (-0.039, 0.073)	0.039 (0.026, 0.052)

[a] Models were adjusted for participants' baseline age, sex, minoritized racial or ethnic groups, paternal educational level, and maternal educational level. A total of 7895 participants were included in CHARLS, 12351 in HRS, 6589 in ELSA, and 9134 in MHAS.

Abbreviations: SD, standard deviation; CI, confidence interval; CHARLS, the China Health and Retirement Longitudinal Study; HRS, the Health and Retirement Study; ELSA, the English Longitudinal Study of Ageing; MHAS, the Mexican Health and Aging Study

eTable 5 The association of maternal education with episodic memory and mental status

	β Coefficients (95% CI)[a]			
	CHARLS	HRS	ELSA	MHAS
**Episodic memory**				
**Maternal educational level, SD**				
< primary	0 [Reference]	0 [Reference]	0 [Reference]	0 [Reference]
primary	0.111 (0.032, 0.190)	0.123 (0.027, 0.219)	-	0.162 (0.123, 0.201)
lower secondary	0.292 (0.060, 0.523)	0.221 (0.122, 0.320)	0.040 (-0.089, 0.168)	0.301 (0.214, 0.387)
≥upper secondary	0.138 (-0.104, 0.381)	0.281 (0.183, 0.378)	0.136 (0.004, 0.267)	-
**Maternal educational level × time, SD/year**				
< primary	0 [Reference]	0 [Reference]	0 [Reference]	0 [Reference]
primary	0.041 (0.017, 0.064)	-0.011 (-0.025, 0.002)	-	0.006 (-0.001, 0.013)
lower secondary	0.054 (-0.022, 0.130)	-0.023 (-0.036, -0.011)	0.006 (-0.013, 0.025)	0.007 (-0.010, 0.024)
≥upper secondary	0.124 (0.053, 0.195)	-0.009 (-0.021, 0.003)	0.015 (-0.004, 0.034)	-
**Mental status**				
**Maternal educational level, SD**				
< primary	0 [Reference]	0 [Reference]	0 [Reference]	0 [Reference]
primary	0.114 (0.056, 0.173)	-0.013 (-0.108, 0.083)	-	0.213 (0.175, 0.251)
lower secondary	0.083 (-0.104, 0.269)	0.043 (-0.055, 0.140)	0.028 (-0.150, 0.205)	0.490 (0.398, 0.581)
≥upper secondary	0.269 (0.102, 0.435)	0.102 (0.007, 0.198)	0.128 (-0.054, 0.309)	-
**Maternal educational level × time, SD/year**				
< primary	0 [Reference]	0 [Reference]	0 [Reference]	0 [Reference]
primary	0.019 (0.004, 0.034)	0.023 (0.007, 0.039)	-	0.010 (0.003, 0.017)
lower secondary	0.067 (0.028, 0.106)	0.040 (0.025, 0.056)	0.027 (0.003, 0.051)	-0.003 (-0.020, 0.014)

© 2025 Luo S et al. *JAMA Network Open*.

≥upper secondary	0.035 (-0.006, 0.077)	0.038 (0.023, 0.053)	0.040 (0.015, 0.064)	-

[a] Models were adjusted for participants' baseline age, sex, minoritized racial or ethnic groups, and paternal educational level; A total of 7898 participants with complete data on covariates were included in CHARLS, 12402 in HRS, 6603 in ELSA, and 9162 in MHAS.

Abbreviations: SD, standard deviation; CI, confidence interval; CHARLS, the China Health and Retirement Longitudinal Study; HRS, the Health and Retirement Study; ELSA, the English Longitudinal Study of Ageing; MHAS, the Mexican Health and Aging Study

© 2025 Luo S et al. *JAMA Network Open*.

eTable 6 The association of paternal education with episodic memory and mental status

	β Coefficients (95% CI)[a]			
	**CHARLS**	**HRS**	**ELSA**	**MHAS**
	**Episodic memory**			
**Paternal educational level, SD**				
< primary	0 [Reference]	0 [Reference]	0 [Reference]	0 [Reference]
primary	0.131 (0.090, 0.172)	0.187 (0.093, 0.282)	-	0.164 (0.125, 0.204)
lower secondary	0.112 (0.000, 0.224)	0.242 (0.144, 0.340)	0.030 (-0.146, 0.206)	0.329 (0.256, 0.401)
≥upper secondary	0.204 (0.084, 0.323)	0.329 (0.232, 0.426)	0.114 (-0.064, 0.293)	-
**Paternal educational level × time, SD/year**				
< primary	0 [Reference]	0 [Reference]	0 [Reference]	0 [Reference]
primary	0.020 (0.007, 0.033)	-0.013 (-0.026, -0.001)	-	0.006 (-0.001, 0.014)
lower secondary	0.025 (-0.008, 0.059)	-0.017 (-0.030, -0.005)	0.022 (-0.003, 0.048)	0.011 (-0.004, 0.025)
≥upper secondary	0.046 (0.012, 0.079)	-0.006 (-0.017, 0.006)	0.028 (0.003, 0.054)	-
	**Mental status**			
**Paternal educational level, SD**				
< primary	0 [Reference]	0 [Reference]	0 [Reference]	0 [Reference]
primary	0.190 (0.156, 0.224)	0.105 (0.015, 0.195)	-	0.166 (0.128, 0.204)
lower secondary	0.165 (0.080, 0.250)	0.091 (-0.001, 0.184)	0.168 (-0.050, 0.385)	0.492 (0.413, 0.570)
≥upper secondary	0.251 (0.162, 0.341)	0.193 (0.103, 0.284)	0.256 (0.036, 0.476)	-
**Paternal educational level × time, SD/year**				
< primary	0 [Reference]	0 [Reference]	0 [Reference]	0 [Reference]
primary	0.013 (0.003, 0.022)	0.026 (0.011, 0.041)	-	0.010 (0.003, 0.017)
lower secondary	0.005 (-0.016, 0.027)	0.047 (0.033, 0.062)	0.029 (-0.004, 0.062)	-0.007 (-0.021, 0.008)
≥upper secondary	0.028 (0.007, 0.050)	0.038 (0.023, 0.052)	0.040 (0.007, 0.074)	-

© 2025 Luo S et al. *JAMA Network Open*.

[a] Models were adjusted for participants' baseline age, sex, minoritized racial or ethnic groups, and maternal educational level; A total of 7898 participants with complete data on covariates were included in CHARLS, 12402 in HRS, 6603 in ELSA, and 9162 in MHAS.

Abbreviations: SD, standard deviation; CI, confidence interval; CHARLS, the China Health and Retirement Longitudinal Study; HRS, the Health and Retirement Study; ELSA, the English Longitudinal Study of Ageing; MHAS, the Mexican Health and Aging Study

© 2025 Luo S et al. *JAMA Network Open*.

eTable 7 Comparison of baseline characteristics between the analytic and excluded participants in CHARLS

	Analytic	Excluded	*p*
N	7898	9810	
Age (years), mean (SD)	60.98 (7.38)	57.32 (11.77)	<0.001
Sex, n (%)			<0.001
Male	4141 (52.4)	4313 (44.0)	
Female	3757 (47.6)	5494 (56.0)	
Minoritized racial or ethnic groups, n (%)			<0.001
Yes	550 (7.0)	709 (8.3)	
No	7348 (93.0)	7796 (91.7)	
Own educational level, n (%)			<0.001
<Primary	1793 (22.7)	3015 (30.8)	
Primary	3552 (45.0)	3406 (34.8)	
Lower secondary	1552 (19.7)	2103 (21.5)	
Upper secondary	840 (10.6)	979 (10.0)	
Postsecondary	158 (2.0)	273 (2.8)	
Maternal educational level, n (%)			<0.001
<Primary	7204 (91.2)	7043 (86.8)	
Primary	592 (7.5)	904 (11.1)	
Lower secondary	52 (0.7)	108 (1.3)	
Upper secondary or higher	50 (0.6)	62 (0.8)	
Paternal educational level, n (%)			<0.001
<Primary	4693 (59.4)	4695 (59.7)	
Primary	2688 (34.0)	2472 (31.4)	
Lower secondary	275 (3.5)	407 (5.2)	
Upper secondary or higher	242 (3.1)	289 (3.7)	

Abbreviations: CHARLS, the China Health and Retirement Longitudinal Study; SD, standard deviation.

© 2025 Luo S et al. *JAMA Network Open*.

eTable 8 Comparison of baseline characteristics between the analytic and excluded participants in HRS

	Analytic	Excluded	*p*
N	12402	9632	
Age (years), mean (SD)	64.85 (9.66)	66.99 (14.33)	<0.001
Sex, n (%)			0.003
Male	5304 (42.8)	3927 (40.8)	
Female	7098 (57.2)	5705 (59.2)	
Minoritized racial or ethnic groups, n (%)			<0.001
Yes	2743 (22.1)	3335 (34.8)	
No	9659 (77.9)	6238 (65.2)	
Own educational level, n (%)			
<Primary	30 (0.2)	129 (1.3)	<0.001
Primary	361 (2.9)	753 (7.9)	
Lower secondary	508 (4.1)	1049 (11.0)	
Upper secondary	4560 (36.9)	4346 (45.4)	
Postsecondary	6892 (55.8)	3297 (34.4)	
Maternal educational level, n (%)			<0.001
<Primary	408 (3.3)	449 (7.3)	
Primary	1550 (12.5)	1090 (17.8)	
Lower secondary	2459 (19.8)	1280 (20.9)	
Upper secondary or higher	7985 (64.4)	3293 (53.9)	
Paternal educational level, n (%)			<0.001
<Primary	425 (3.4)	371 (8.1)	
Primary	2110 (17.0)	1008 (22.1)	
Lower secondary	2800 (22.6)	976 (21.4)	
Upper secondary or higher	7067 (57.0)	2203 (48.3)	

Abbreviations: HRS, the Health and Retirement Study; SD, standard deviation.

© 2025 Luo S et al. *JAMA Network Open*.

eTable 9 Comparison of baseline characteristics between the analytic and excluded participants in ELSA

	Analytic	Excluded	*p*
N	6603	3671	
Age (years), mean (SD)	65.29 (8.17)	68.80 (11.56)	<0.001
Sex, n (%)			0.380
Male	2915 (44.1)	1653 (45.1)	
Female	3688 (55.9)	2016 (54.9)	
Minoritized racial or ethnic groups, n (%)			<0.001
Yes	155 (2.3)	198 (5.4)	
No	6448 (97.7)	3464 (94.6)	
Own educational level, n (%)[a]			<0.001
<Primary	19 (0.3)	22 (0.6)	
Primary	-	-	
Lower secondary	1732 (26.3)	1637 (45.4)	
Upper secondary	1605 (24.4)	763 (21.2)	
Postsecondary	3233 (49.1)	1184 (32.8)	
Maternal educational level, n (%)[a]			<0.001
<Primary	91 (1.4)	92 (4.0)	
Primary	-	-	
Lower secondary	4703 (71.2)	1771 (76.1)	
Upper secondary or higher	1809 (27.4)	465 (20.0)	
Paternal educational level, n (%)[a]			<0.001
<Primary	51 (0.8)	65 (3.0)	
Primary	-	-	
Lower secondary	4813 (72.9)	1690 (76.9)	
Upper secondary or higher	1739 (26.3)	443 (20.2)	

[a] Available options of parental and participants' own education in ELSA did not allow for a distinction between primary and lower secondary education.

Abbreviations: ELSA, the English Longitudinal Study of Ageing; SD, standard deviation.

© 2025 Luo S et al. *JAMA Network Open*.

eTable 10 Comparison of baseline characteristics between the analytic and excluded participants in MHAS

	Analytic	Excluded	*p*
N	9162	6561	
Age (years), mean (SD)	63.38 (8.49)	65.66 (13.23)	<0.001
Sex, n (%)			0.520
Male	3863 (42.2)	2733 (41.7)	
Female	5299 (57.8)	3828 (58.3)	
Own educational level, n (%)			<0.001
<Primary	1240 (13.6)	1569 (24.0)	
Primary	4713 (51.6)	3264 (50.0)	
Lower secondary	1737 (19.0)	981 (15.0)	
Upper secondary	452 (4.9)	230 (3.5)	
Postsecondary	993 (10.9)	480 (7.4)	
Maternal educational level, n (%)[a]			<0.001
<Primary	4489 (49.0)	2436 (55.2)	
Primary	4221 (46.1)	1783 (40.4)	
Lower secondary	452 (4.9)	194 (4.4)	
Upper secondary or higher	-	-	
Paternal educational level, n (%)[a]			<0.001
<Primary	3926 (42.9)	1998 (49.6)	
Primary	4556 (49.7)	1719 (42.7)	
Lower secondary	680 (7.4)	308 (7.7)	
Upper secondary or higher	-	-	

[a] Available options of parental education in MHAS did not allow for a distinction between lower secondary and upper secondary education.

Abbreviations: MHAS, the Mexican Health and Aging Study; SD, standard deviation.

© 2025 Luo S et al. *JAMA Network Open*.

eTable 11 The association between maternal education and cognitive function with imputed dataset

	β Coefficients (95% CI)[a]			
	CHARLS	HRS	ELSA	MHAS
**Cognitive state**				
**Maternal educational level, SD**				
< primary	0 [Reference]	0 [Reference]	0 [Reference]	0 [Reference]
primary	0.158 (0.101, 0.215)	0.103 (0.023, 0.182)	-	0.217 (0.183, 0.251)
lower secondary	0.204 (0.033, 0.375)	0.239 (0.156, 0.321)	0.059 (-0.123, 0.242)	0.500 (0.418, 0.582)
≥upper secondary	0.272 (0.090, 0.454)	0.289 (0.208, 0.371)	0.143 (-0.043, 0.329)	-
**Maternal educational level × time, SD/year**				
< primary	0 [Reference]	0 [Reference]	0 [Reference]	0 [Reference]
primary	0.035 (0.019, 0.050)	0.010 (-0.002, 0.022)	-	0.011 (0.005, 0.017)
lower secondary	0.068 (0.024, 0.111)	0.007 (-0.004, 0.018)	0.022 (0.001, 0.043)	-0.003 (-0.018, 0.013)
≥upper secondary	0.082 (0.031, 0.133)	0.022 (0.012, 0.032)	0.041 (0.019, 0.063)	-
**Episodic memory**				
**Maternal educational level, SD**				
< primary	0 [Reference]	0 [Reference]	0 [Reference]	0 [Reference]
primary	0.132 (0.061, 0.203)	0.151 (0.067, 0.235)	-	0.159 (0.122, 0.196)
lower secondary	0.264 (0.048, 0.480)	0.263 (0.178, 0.348)	0.048 (-0.113, 0.209)	0.305 (0.224, 0.386)
≥upper secondary	0.221 (-0.011, 0.453)	0.316 (0.231, 0.400)	0.128 (-0.037, 0.292)	-
**Maternal educational level × time, SD/year**				
< primary	0 [Reference]	0 [Reference]	0 [Reference]	0 [Reference]
primary	0.048 (0.026, 0.069)	-0.007 (-0.019, 0.005)	-	0.006 (-0.001, 0.013)
lower secondary	0.063 (-0.007, 0.132)	-0.018 (-0.029, -0.007)	0.007 (-0.013, 0.027)	0.004 (-0.013, 0.021)
≥upper secondary	0.117 (0.046, 0.188)	-0.002 (-0.012, 0.008)	0.022 (0.001, 0.042)	-
**Mental status**				
**Maternal educational level, SD**				
< primary	0 [Reference]	0 [Reference]	0 [Reference]	0 [Reference]
primary	0.133 (0.080, 0.186)	0.038 (-0.041, 0.118)	-	0.208 (0.173, 0.243)
lower secondary	0.118 (-0.045, 0.281)	0.145 (0.064, 0.227)	0.056 (-0.131, 0.244)	0.498 (0.413, 0.583)
≥upper secondary	0.228 (0.071, 0.385)	0.165 (0.084, 0.245)	0.129 (-0.063, 0.320)	-
**Maternal educational level × time, SD/year**				
< primary	0 [Reference]	0 [Reference]	0 [Reference]	0 [Reference]
primary	0.020 (0.006, 0.035)	0.018 (0.005, 0.030)	-	0.011 (0.005, 0.017)
lower secondary	0.055 (0.011, 0.098)	0.021 (0.009, 0.032)	0.024 (0.001, 0.046)	-0.004 (-0.021, 0.012)
≥upper secondary	0.045 (-0.002, 0.093)	0.030 (0.019, 0.041)	0.042 (0.019, 0.065)	-

[a] Models were adjusted for participants' baseline age, sex, minoritized racial or ethnic groups, and paternal educational level.

Abbreviations: SD, standard deviation; CI, confidence interval; CHARLS, the China Health and Retirement Longitudinal Study; HRS, the Health and Retirement Study; ELSA, the English Longitudinal Study of Ageing; MHAS, the Mexican Health and Aging Study

© 2025 Luo S et al. *JAMA Network Open*.

eTable 12 The association between paternal education and cognitive function with imputed dataset

	β Coefficients (95% CI)[a]			
	CHARLS	HRS	ELSA	MHAS
**Cognitive state**				
**Paternal educational level, SD**				
< primary	0 [Reference]	0 [Reference]	0 [Reference]	0 [Reference]
primary	0.210 (0.177, 0.242)	0.176 (0.097, 0.255)	-	0.180 (0.145, 0.214)
lower secondary	0.227 (0.148, 0.306)	0.243 (0.161, 0.326)	0.118 (-0.101, 0.336)	0.495 (0.425, 0.564)
≥upper secondary	0.319 (0.229, 0.409)	0.334 (0.253, 0.416)	0.184 (-0.038, 0.406)	-
**Paternal educational level × time, SD/year**				
< primary	0 [Reference]	0 [Reference]	0 [Reference]	0 [Reference]
primary	0.021 (0.012, 0.030)	0.012 (-0.0005, 0.023)	-	0.009 (0.004, 0.015)
lower secondary	0.012 (-0.010, 0.034)	0.015 (0.004, 0.026)	0.027 (-0.002, 0.056)	-0.002 (-0.014, 0.010)
≥upper secondary	0.046 (0.025, 0.067)	0.022 (0.011, 0.033)	0.043 (0.013, 0.072)	-
**Episodic memory**				
**Paternal educational level, SD**				
< primary	0 [Reference]	0 [Reference]	0 [Reference]	0 [Reference]
primary	0.132 (0.093, 0.171)	0.169 (0.085, 0.252)	-	0.164 (0.127, 0.202)
lower secondary	0.156 (0.054, 0.258)	0.242 (0.155, 0.329)	0.091 (-0.115, 0.296)	0.347 (0.280, 0.415)
≥upper secondary	0.263 (0.149, 0.377)	0.311 (0.226, 0.396)	0.155 (-0.053, 0.363)	-
**Paternal educational level × time, SD/year**				
< primary	0 [Reference]	0 [Reference]	0 [Reference]	0 [Reference]
primary	0.025 (0.013, 0.037)	-0.007 (-0.018, 0.004)	-	0.006 (-0.001, 0.013)
lower secondary	0.022 (-0.011, 0.056)	-0.011 (-0.021, 0.000)	0.022 (-0.004, 0.049)	0.009 (-0.004, 0.023)
≥upper secondary	0.050 (0.018, 0.081)	0.002 (-0.008, 0.012)	0.032 (0.005, 0.059)	-
**Mental status**				
**Paternal educational level, SD**				
< primary	0 [Reference]	0 [Reference]	0 [Reference]	0 [Reference]
primary	0.206 (0.175, 0.238)	0.120 (0.043, 0.197)	-	0.164 (0.129, 0.200)
lower secondary	0.213 (0.139, 0.288)	0.162 (0.082, 0.242)	0.119 (-0.105, 0.343)	0.481 (0.409, 0.553)
≥upper secondary	0.275 (0.191, 0.359)	0.223 (0.143, 0.302)	0.175 (-0.052, 0.402)	-
**Paternal educational level × time, SD/year**				
< primary	0 [Reference]	0 [Reference]	0 [Reference]	0 [Reference]
primary	0.015 (0.006, 0.023)	0.019 (0.007, 0.032)	-	0.009 (0.003, 0.016)
lower secondary	0.004 (-0.017, 0.024)	0.026 (0.014, 0.038)	0.025 (-0.006, 0.057)	-0.005 (-0.018, 0.008)
≥upper secondary	0.035 (0.013, 0.057)	0.027 (0.015, 0.039)	0.041 (0.009, 0.072)	-

[a] Models were adjusted for participants' baseline age, sex, minoritized racial or ethnic groups, and maternal educational level.

Abbreviations: SD, standard deviation; CI, confidence interval; CHARLS, the China Health and Retirement Longitudinal Study; HRS, the Health and Retirement Study; ELSA, the English Longitudinal Study of Ageing; MHAS, the Mexican Health and Aging Study

© 2025 Luo S et al. *JAMA Network Open*.

eReferences

1. Glosser G, Wolfe N, Albert ML, et al. Cross-cultural cognitive examination: validation of a dementia screening instrument for neuroepidemiological research. *J Am Geriatr Soc*. 1993;41(9):931-9. doi:10.1111/j.1532-5415.1993.tb06758.x
2. Lamberty GJ, Kennedy CM, Flashman LA. Clinical utility of the CERAD word list memory test. *Appl Neuropsychol*. 1995;2(3-4):170-3. doi:10.1080/09084282.1995.9645357
3. Herzog AR, Wallace RB. Measures of cognitive functioning in the AHEAD Study. *J Gerontol Ser B-Psychol Sci Soc Sci*. 1997;52(Special_Issue):37-48.
4. Sebaldt R, Dalziel W, Massoud F, et al. Detection of cognitive impairment and dementia using the animal fluency test: the DECIDE study. *Can J Neurol Sci*. 2009;36(5):599-604. doi:10.1017/s0317167100008106
5. Folstein MF, Folstein SE, McHugh PR. "Mini-mental state". A practical method for grading the cognitive state of patients for the clinician. *J Psychiatr Res*. 1975;12(3):189-98. doi:10.1016/0022-3956(75)90026-6

© 2025 Luo S et al. *JAMA Network Open*.
