# Supplementary material for: Parental Education, Own Education, and Cognitive Function in Middle-Aged and Older Adults
Source: JAMA Netw Open. 2025 May 30;8(5):e2513036. doi: 10.1001/jamanetworkopen.2025.13036 (PMC12125642; doi:10.1001/jamanetworkopen.2025.13036)
Supplement: Supplement 2. — Data Sharing Statement [file jamanetwopen-e2513036-s002.pdf]

Data Sharing Statement

Luo. Parental Education, Own Education, and Cognitive Function in Middle-Aged and Older Adults. *JAMA Netw Open*. Published May 30, 2025. doi:10.1001/jamanetworkopen.2025.13036

Data
Data available: Yes
Data types: Deidentified participant data
How to access data: The CHARLS datasets were available at http://charls.pku.edu.cn/en/. The HRS datasets can be accessed at https://hrs.isr.umich.edu/. The ELSA datasets were available from the UK Data Service at https://ukdataservice.ac.uk/. The MHAS datasets were available at https://www.mhasweb.org/DataProducts/Home.aspx.
When available: With publication

Supporting Documents
Document types: None

Additional Information
Who can access the data: Data from the China Health and Retirement Longitudinal Study (CHARLS),the Health and Retirement Study (HRS), the English Longitudinal Study of Ageing (ELSA), and the Mexican Health and Aging Study (MHAS) are available to all researchers upon making an application.
Types of analyses: All types of analyses with an application approved.
Mechanisms of data availability: After approval of an application.
